# Lemongrass Alleviates Primary Dysmenorrhea Symptoms by Reducing Oxidative Stress and Inflammation and Relaxing the Uterine Muscles

**DOI:** 10.3390/antiox14070838

**Published:** 2025-07-08

**Authors:** Sheikh Safeena Sidiq, Qaiser Jabeen, QurratUlAin Jamil, Muhammad Saeed Jan, Iram Iqbal, Fatima Saqib, Mohammed Aufy, Shahid Muhammad Iqbal

**Affiliations:** 1 Department of Pharmacology, Faculty of Pharmacy, The Islamia University of Bahawalpur, Bahawalpur 63100, Pakistan; safeena.sidiq@iub.edu.pk (S.S.S.);; 2 Department of Pharmacy Practice, Faculty of Pharmacy, The Islamia University of Bahawalpur, Bahawalpur 63100, Pakistan; 3 Department of Pharmacy, Bacha Khan University, Charsadda 24420, Pakistan; 4 Department of Pharmacology, Faculty of Pharmacy, Bahauddin Zakariya University, Multan 60800, Pakistan; 5 Division of Pharmacology and Toxicology, University of Vienna, UZA II, Josef-Holaubek-Platz 2, A-1090 Vienna, Austria; 6 Michael Sars Center, University of Bergen, 5007 Bergen, Norway

**Keywords:** lemongrass, dysmenorrhea, antioxidant, tocolytic, analgesic, anti-inflammatory

## Abstract

Primary dysmenorrhea (PD) is characterized by lower abdominal spasms and painful cramps during menstruation in females with a normal pelvic anatomy. *Cymbopogon citratus* (DC.) Stapf, commonly known as lemongrass, is consumed in the form of herbal tea around the world. It has been traditionally used for menstrual disorders in several communities. This study aims to evaluate the traditional use of *C. citratus* for its efficacy in alleviating the symptoms of PD. *C. citratus* extract (CcE) was chemically characterized using HPLC and GCMS, which indicated the presence of several phenolic compounds and long-chain fatty acids. The anti-inflammatory activity of CcE was assessed by COX-I, COX-II, and 5-LOX enzyme inhibition with IC_50_ values of 143.7, 91.7, and 61.5 µg/mL, respectively, and showed good total antioxidant capacity and free radical scavenging activity. PD was induced in female Wistar rats by administering estradiol valerate followed by oxytocin to induce PD symptoms. CcE efficacy was assessed at 30, 100, and 300 mg/kg concentrations and compared with ibuprofen. CcE 300 mg/kg reduced abdominal contortions and inflammation in the rat uterus. The inflammatory (COX-II, TNFα and IL-10) and oxidative stress (TAC, TOS, MDA and SOD) markers in uterine tissue homogenate were also improved. An in vivo analgesic assessment through hot-plate, tail-flick, and acetic acid-induced writhing assays showed good analgesic activity by CcE, while ex vivo experiments described tocolytic effects in rat uterine muscles. CcE alleviates PD by its antioxidant, anti-inflammatory, analgesic, and tocolytic effects.

## 1. Introduction

The female reproductive system undergoes periodic cyclic changes to prepare for pregnancy and fertilization, called the menstrual cycle. It is characterized by monthly vaginal bleeding with the shedding of uterine mucosa (menstruation). The length of this cycle normally varies between 21 and 35 days, with an average duration of 28 days, and lasts from the first day of menstrual bleeding to the start of the next cycle, while the duration of bleeding ranges from 2 to 7 days [1]. This cyclic process is finely regulated by stimulating or inhibiting endocrine, paracrine, and autocrine factors at five different levels, including the cerebral cortex, hypothalamus, pituitary glands, ovaries, and uterus [2].

Primary dysmenorrhea (PD) is a common gynecologic condition characterized by lower abdominal cramps starting with the onset of menstruation without any pelvic pathology that persists for 2–3 days [3]. It is categorized into primary and secondary dysmenorrhea, with the latter having an underlying pelvic pathology. Its prevalence ranges between 42 and 89% around the globe [4]. This painful menstruation is quite disabling and has a negative impact on quality of life, including personal, social, and family life; relationships; school/work performance; and recreational activities. This intense cyclic pain causes limitations in physical activity, and can result in absence from school or work, affecting productivity and sleep disturbance, and inducing emotional and psychological distress [5]. Many women do not seek medical attention, considering pain to be a symptom accompanying menstruation, and use complementary and alternative measures for pain relief. The pathogenesis of PD mainly involves increased prostaglandin production in uterine tissue, causing contractions and ischemia with increased nerve sensitivity. Several other factors, including leukotrienes, vasopressin, increased basal uterine tone, oxidative stress, oxytocin, sex hormones, vasopressin, and β-endorphins, also contribute to the etiology of PD. Its management includes non-steroidal anti-inflammatory drugs (NSAIDs), calcium antagonists, and contraceptives, which alleviate the symptoms variably [6]. Approximately 47–70% of adolescents use analgesics for pain relief, with 30% using over-the-counter (OTC) and 18% using prescription medicines. In a study involving 289 female participants, approximately 98% reported the use of non-pharmacologic measures for pain relief, with a perceived effectiveness of 40% [5,7]. Non-pharmacological options are used to complement the first-line therapies. Several studies have reported the effectiveness of aromatic oils, tea, rose tea, fenugreek, ginger, fennel, and Chinese herbal medicines in PD [8].

*Cymbopogon citratus* (DC.) Stapf, known as lemongrass, belongs to the family Poaceae, found in China, Pakistan, India, Africa, America, and several other countries. It is a perennial grass with scented leaves and reaches a height of one meter [9]. *C. citratus* is the most cultivated plant of the genus *Cymbopogon*, and, in several communities, it is consumed as a popular, pleasant, flavored herbal tea [10]. Economically, it is an important plant and widely used for phytotherapy, pharmaceuticals, the food industry, and in cosmetics [10]. It contains several medicinally important phytochemicals [9,11] and is used as an antioxidant, anti-inflammatory, analgesic, antimicrobial, antidiarrheal, hypoglycemic, and hypolipidemic [12]. Traditionally, *C. citratus* is used by several communities as an emmenagogue and to relieve pain in dysmenorrhea [10,12,13]. Since it is known for its antioxidant, anti-inflammatory, analgesic, antispasmodic, and vasorelaxant activities [13,14,15], we aimed to evaluate its efficacy in relieving the inflammation, oxidative stress, and menstrual pain in PD.

## 2. Materials and Methods

### 2.1. Preparation of CcE

Aerial parts of *C. citratus* (DC.) Stapf (2 kg) were collected from the garden of Khawaja Farid Campus, Bahawalpur (29.395840, 71.661772), and identified by Dr. Ghulam Sarwar, Department of Botany, the Islamia University of Bahawalpur. The plant material specimen has a voucher no. 241 and was kept in the herbarium of Pharmacology Research Lab, Faculty of Pharmacy, the Islamia University of Bahawalpur. The collected plant material was cleaned, shade-dried, and then coarsely pulverized. *C. citratus* (500 g) was extracted in methanol (70%) for three days in a plastic jar with daily shaking. After three days, the macerate was initially double-filtered through muslin cloth and then through filter paper. This process of extraction was repeated twice, and the total filtrate was collected and evaporated on a rotary evaporator (40 °C; 150 mbar) until it was concentrated into a semi-solid mass. The percentage yield of *C. citratus* extract (CcE) was calculated and stored at −20 °C. Fractions were prepared by extracting *C. citratus* (100 g dried plant) in n-hexane, n-butanol, ethyl acetate, methanol, and water by following the same procedure as described for CcE.

### 2.2. Phytochemical Analysis

CcE was qualitatively assayed for the presence of carbohydrates, glycosides, alkaloids, tannins, flavonoids, and saponins as previously described [16].

#### 2.2.1. Total Phenolic Contents

Total phenolic contents (TPCs) were determined as previously described [17]. The 50 µL of diluted Folin–Ciocalteu (FC) reagent (1:10) was vortexed with 50 µL of CcE (1 mg/mL) or gallic acid (0.007–1.0 mg/mL). Afterwards, 500 µL of Na_2_CO_3_ (7%) and 650 µL of distilled water (dH_2_O) were added, and the mixture was incubated for 1 h in the dark. Absorbance was recorded at 750 nm, and TPC was calculated as mg of gallic acid equivalents per gram of CcE (mg GAE/g CcE).

#### 2.2.2. Total Tannin Contents

Total tannins in CcE were determined as previously described, with minor modifications [16]. A standard curve of tannic acid (0.1–1.0 mg/mL) was constructed. Tannins in 1 mL CcE (100 mg/mL) were precipitated by adding polyvinylpolypyrrolidone (100 mg) and 1 mL dH_2_O. The mixture was vortexed and incubated at 4 °C for 4 h. After incubation, the reaction mixture was centrifuged at 3000 rpm for 10 min, and the supernatant was collected. TPC before and after precipitation of tannins were determined by FC reagent and expressed as mg tannic acid equivalent per gram of CcE (mg TAE/g CcE).

#### 2.2.3. Total Flavonoid Contents

The flavonoid contents were determined as previously described [18]. The reaction mixture consisted of 125 µL CcE (1 mg/mL) or quercetin (7.8–500 µg/mL), 200 µL of dH_2_O, and 45 µL of sodium nitrite (5%), and was incubated for 5 min at room temperature. Afterwards, 90 µL of aluminum chloride (10%) was added, and the mixture was again incubated for 5 min. Then, 300 µL sodium hydroxide (1 N) and 740 µL of deionized water were added, with adjustment of final volume to 1.5 mL. After a 15 min final incubation, absorbance was recorded at 510 nm. TFC were calculated as mg quercetin equivalents per gram of CcE (mg QE/g CcE).

#### 2.2.4. Total Saponin Contents

Total saponin contents (TSCs) were determined by taking 20 µL of CcE (10 mg/mL), 100 µL dH_2_O, 100 µL vanillin (8% in 99.5% ethanol), and 1 mL of sulfuric acid (72%). The reaction mixture was vortexed and incubated at 60 °C for 20 min. After incubation, the sample was rapidly cooled using ice-cold water, and absorbance was measured at 544 nm. TSC were calculated as mg diosgenin equivalents per gram of CcE (mg DE/g CcE) [19].

### 2.3. HPLC Analysis

CcE phenolic compounds were quantified as described previously [20]. CcE (50 mg) was homogenized in methanol (24 mL), followed by the addition of 6M HCl (10 mL) and dH_2_O (16 mL). The mixture was refluxed for 2 h at 95 °C, followed by filtration (0.45 µm). The prepared sample was separated on HPLC (Shimadzu, Kyoto, Japan) by using a reversed-phase C_18_ column (4.6 × 250 mm; 5 µm) with a mobile phase consisting of acetonitrile and a mixture of HPLC-grade water and acetic acid (94:6, pH 2.2). The gradient was created with 15% acetonitrile (0–15 min), 45% acetonitrile (15–30 min), and 100% acetonitrile (30–45 min), keeping the flow rate 1 mL/min. For flavonoids, the mobile phase consisted of acetonitrile, dichloromethane, and methanol (60:20:20), with a flow rate of 0.5 mL/min. Detection was performed by using UV at 248 or 280 nm, while identification was achieved by comparing the peaks and retention times of the standards.

### 2.4. GC-MS Analysis

GC-MS analysis of CcE was performed as described previously [16] by using a Thermo Fisher Scientific GC (Waltham, MA, USA). A TR-5MS capillary column (30 m × 0.25 mm ID and 0.25 µm film thickness) was used, with helium (1 mL/min) serving as a carrier gas. To detect GC-MS spectra, an ionization energy of 70 electron volts was applied for 0.2 s with a mass range of 40–600 *m*/*z*. A split-mode injector was operated at 250 °C. The CcE sample (1 µL) was injected, with the oven temperature set at 50 °C for 2 min initially, then gradually increased (8 °C/min) to 150 °C, followed by an increase to 300 °C at 15 °C/min, and maintained at 300 °C for 5 min. The chemical constituents within the plant extract were identified based on the retention time, peak area, peak height, and spectral pattern from the NIST11.L library.

### 2.5. Antioxidant Assays

#### 2.5.1. DPPH Assay

The 2,2-diphenyl-1-picrylhydrazyl (DPPH) radical scavenging activity of CcE was assessed by a previously established method with minor modification [21]. The assay was performed by adding 50 μL of various concentrations of ascorbic acid (0.001–1.0 mg/mL) or CcE (0.001–5.0 mg/mL) in a 96-well plate. Subsequently, 150 μL of DPPH (0.2 mM) was added. The plate was incubated in the dark for 30 min, followed by reading the absorbance at 517 nm, with 95% methanol serving as a blank. The control consisted of 50 μL of methanol instead of the sample. The given formula was used to calculate the percentage inhibition.

$$\%\;\mathrm{DPPH}\;\mathrm{scavenging}=[1-\left(\frac{\mathrm{Asample}-\mathrm{Ablank}}{\mathrm{Acontrol}-\mathrm{Ablank}}\;\right)]\times 100$$

#### 2.5.2. Nitric Oxide Scavenging Assay

The NO scavenging assay was performed as described previously [21]. CcE (0.01–2.0 mg/mL) and ascorbic acid (0.007–1.0 mg/mL) were prepared. The CcE/ascorbic acid 100 µL was combined with 100 µL of NaNO_2_ (1 mM; pH 2). The reaction mixture was then diluted to 1 mL with dH_2_O and incubated for 1 h at 37 °C. After incubation, 40 µL of Griess reagent and 200 µL of water were added to 40 µL of each reaction mixture, which was then incubated at room temperature for 15 min. Absorbance was recorded in the presence and absence of Griess reagent, at 540 nm with dH_2_O as a control. The % scavenging activity was calculated by the given formula:

$$\%\;\mathrm{scavenging}\;\mathrm{activity}=100-\left[\left\{\frac{\mathrm{Abs}.\;\mathrm{with}\;\mathrm{GR}-\mathrm{Abs}.\;\mathrm{without}\;\mathrm{GR}}{\mathrm{Abs}.\;\mathrm{of}\;\mathrm{Control}}\right\}\times 100\right]$$

#### 2.5.3. CUPRAC Assay

The cupric reducing antioxidant capacity (CUPRAC) assay was performed by the previously described method with minor modifications [21]. Using CcE (0.039–5.0 mg/mL) or ascorbic acid (0.007–1.0 mg/mL), 200 μL was added to each well, while the blank contained solvent only. Absorbance was measured at 450 nm before the addition of the CUPRAC reagent. Afterwards, 50 µL of the CUPRAC reagent was added and incubated at room temperature for 15 min. The reaction was terminated by adding 50 µL of EDTA-Na_2_ (20 mM), and the final absorbance was measured at 450 nm. The EC_50_ value was calculated by normalizing the net absorbance and plotting it against the concentrations.

#### 2.5.4. H_2_O_2_ Scavenging Assay

The H_2_O_2_ scavenging assay was performed according to a previously described method with minor modifications [22]. The control tube contained 100 µL phosphate-buffered saline (PBS; pH 7.4), 80 µL H_2_O_2_ (2 mM), 40 µL dH_2_O, and 80 µL CuCl_2_·2H_2_O (0.1 mM). In two additional sets, 100 µL of PBS (pH 7.4), 80 µL of H_2_O_2_ (2 mM), 40 µL of sample or standard solution, and 80 µL of CuCl_2_·2H_2_O (0.1 mM) were added sequentially. The mixtures were incubated at 37 °C for 30 min. After incubation, 80 µL of water was added to the first set of sample/standard solutions. To the second set of sample/standard solutions, 80 µL of 268 UmL^−1^ catalase solution was added, and vortexed. To 50 µL of each reaction mixture, 200 µL of CUPRAC reagent was added. After 30 min of incubation, absorbance was measured at 450 nm against a reagent blank. The given formula calculated the hydroxyl peroxide scavenging (HPS) activity:

$$\mathrm{HPS}\;(\%)=100[\frac{A0-\left(A1-A2\right)}{A0}]$$

A0 = CUPRAC absorbance of the reference H_2_O_2_ incubation solution; A1 = CUPRAC absorbance of solution I; A2 = CUPRAC absorbance of solution II.

#### 2.5.5. FRAP Scavenging Assay

The reducing power of CcE was measured as described previously [23]. Briefly, 100 µL of CcE or butylated hydroxytoluene (0.125–2.0 mg/mL) was mixed with 250 µL of PBS (0.2 M; pH 6.6), and 250 µL of potassium ferricyanide (10 mg/mL). The mixture was incubated at 50 °C for 30 min, followed by the addition of 250 µL of TCA (100 mg/mL), and then centrifuged at 3000 rpm for 10 min. The supernatant (125 µL) was collected and mixed with 125 µL of dH_2_O, followed by the addition of 50 µL of FeCl_3_ solution (1 mg/mL). Absorbance was measured at 700 nm. EC_50_ was calculated from the normalized absorbance.

### 2.6. In Vitro Anti-Inflammatory Activity

#### 2.6.1. Cyclooxygenase Inhibition

COX-I and COX-II enzyme inhibitory assays were performed by the previously described method [21]. A total of 10 µL of COX-1 (0.7–0.8 µg) or COX-2 (300 U/mL) was activated on ice for 5–10 min by adding 50 µL of hematin (1 mM). The enzyme solution contained tetramethylphenylenediamine (0.24 mM) and GSH (0.9 mM) in Tris-HCl buffer (0.1 M, pH 8.0). This activated enzyme mixture was added to 20 µL of CcE/fraction dilutions (31.25–500 µg/mL) or indomethacin/celecoxib and incubated at room temperature for 5 min. To start the reaction, 20 µL of arachidonic acid (30 mM) was added and incubated at room temperature for 15 min. HCl was added to stop the reaction, and absorbance was measured at 570 nm.

#### 2.6.2. Lipoxygenase Inhibition

The 5-LOX inhibition assay was performed as described previously [21]. A total of 250 µL of CcE/fraction dilutions (31.25–500 µg/mL) or montelukast was added to an equal volume of 5-LOX (10,000 U/mL) enzyme and incubated at room temperature for 5 min. After that, 1 mL of linoleic acid (0.6 mM) was added, and absorbance was measured at 234 nm. The IC_50_ was calculated from the concentration–response curve.

### 2.7. Animals

Non-pregnant female Wistar albino rats (weighing 130–175 g) and Swiss albino mice (weighing 15–30 g) were used for the experimental study. The animals were housed in the animal care facility under the standard conditions described previously [24]. The experimental protocols were reviewed and approved by the Pharmacy Animal Ethics Committee of the Islamia University of Bahawalpur (PAEC/24/112). Before ex vivo experiments, female Wistar rats were treated with estradiol valerate (8 mg/kg/day) for two consecutive days to align their estrous cycle. Overnight fasted animals were sacrificed by cervical dislocation, and uterine muscle tissue was excised and kept in a pre-warmed Locke’s solution (37 °C), aerated with 5% CO_2_ and 95% CO_2_.

#### 2.7.1. Acute Toxicity Study

Acute oral toxicity of CcE was assessed in mice by following the OECD 425 guidelines [16]. Initially, one animal was administered 2 g/kg CcE orally and monitored for 30 min, then at 4 and 24 h for any signs of toxicity. If no toxicity appeared, then four additional animals were administered 2 g/kg CcE and monitored for 14 consecutive days for any signs of toxicity or altered behavior.

#### 2.7.2. Induction of PD and Treatments

PD was induced in female Wistar rats. Animals were divided into six groups, including normal, PD, CcE 30, 100, 300 mg/kg, and Ibuprofen 100 mg/kg. Estradiol valerate was administered intragastrically for 12 days (5 mg/kg on the 1st and 12th day, with 3 mg/kg from the 2nd to 11th day), followed by i.p. oxytocin (20 U/kg) on the 13th day to induce the primary dysmenorrhea pain. Animals were treated according to their designated groups starting from the 6th day of the model. On the 13th day after oxytocin administration, writhing latency and the total number of writhes were recorded for 30 min. The criteria for counting a writhing response included abdominal recession, the front abdominal wall contacting the cage bottom, twisting of the hips, and stretching of the hind limbs. After recording writhes, animals were euthanized, and blood was collected into two tubes, one containing EDTA for hematological analysis and the other for serum collection. Hematological parameters were assessed using a fully automated hematology analyzer. The blood was centrifuged at 7000 rpm for 20 min to collect the serum, which was then stored at −20 °C. Morphometric assessment of the uterine tissue was performed before excision, measuring the size from the base of the uterine horn to the ovary, followed by measuring its weight and edema [6]. A part of the uterine tissue was fixed in formalin (10%) for histopathology. After dehydration, the paraffin-embedded tissue was sectioned into 4 μm slices and stained with hematoxylin and eosin (H&E). The pathological morphology of the uterine tissue was observed at 200× and 400× magnification using an Olympus BX53 upright microscope (Olympus, Tokyo, Japan). Neutrophil infiltration, edema, endometrial hyperplasia, and myometrial thickness were observed. The remaining tissue was homogenized and stored at −20 °C for analysis of biochemical parameters including malondialdehyde (MDA), superoxide dismutase (SOD), catalase (CAT), glutathione (GSH), total oxidative stress (TOS), total antioxidant capacity (TAC), and nitric oxide (NO) by using the commercial kits. Inflammatory markers from uterine tissue homogenate, including COX-II, TNFα, IL1β, IL10, PGI2, TXA2, and hormones such as estrogen and progesterone from serum, were assessed using commercially available ELISA kits.

#### 2.7.3. Ex Vivo Uterine Tissue Relaxant Activity

The excised uterine muscle tissue was attached to a transducer with 1 g baseline tension and equilibrated for 30 min. CcE was added in a cumulative manner (0.01–10 mg/mL) to check the effect on the basal tone of the uterine tissue. In another set of experiments, after equilibration, uterine tissue was treated with high KCl (80 mM), which induced sustained contractions and the relaxant effect of CcE (0.01–10 mg/mL) was assessed. To check the effect of CcE on oxytocin-induced contractions, uterine tissue was contracted twice by oxytocin (0.008 U/mL), and CcE was added cumulatively (0.01–10 mg/mL) to check the relaxant effect. In all experiments, verapamil was used as a standard.

#### 2.7.4. Analgesic Activity

Analgesic activity was performed on Swiss albino mice according to the previously described procedures [24]. Animals were randomly assigned into five groups: normal control, ibuprofen (10 mg/kg), and CcE30, 100, and 300 mg/kg.

##### Hot-Plate Activity

The hot plate was adjusted to 55 ± 1 °C. Animals were placed on the hot plate, and the time between the placement and the occurrence of nociceptive responses, including jumping, paw withdrawal, and paw licking, was recorded in seconds. Mice with a reaction time of ≥10 s were excluded from the study, while the cut-off time was kept at 20 s. Responses were measured at 0, 30 min, 1, 2, 3, and 4 h after the administration of the CcE or ibuprofen.

##### Tail-Flick Activity

Tail-flick activity was performed by immersing the animal’s tail up to 3 cm in hot water maintained at 55 ± 1 °C. Animals with response times ≥10 s with no treatment were excluded from the study, while a cutoff time of 20 s was set to prevent any potential harm to animals. The latency time of tail withdrawal was recorded.

##### Acetic Acid-Induced Writhing

Animals were pre-treated with saline, CcE, or ibuprofen for 30 min, followed by the administration of 10 mL/kg i.p. acetic acid (0.7%). Animals were placed into a polypropylene cage, and the total number of stretches and writhes was recorded for 30 min. The percentage inhibition for each treatment was calculated.

### 2.8. Statistical Analysis

Data was presented as mean ± SEM. Significance was measured by ANOVA followed by a suitable post hoc test by GraphPad Prism 8. Statistical significance was considered at *p* ≤ 0.05.

## 3. Results

### 3.1. CcE Extraction and Phytochemical Analysis

The highest yield of 17.7% was obtained with 70% methanol and named CcE. While the yields with methanol, n-butanol, ethyl acetate, and n-hexane were 11.7%, 11.0%, 5.6%, and 3.2% respectively. A qualitative phytochemical analysis of CcE indicated the presence of alkaloids, flavonoids, tannins, phenols, glycosides, terpenes, coumarins, quinones, and saponins. CcE contained total phenolic contents of 1.75 ± 0.03 mg GAE/g CcE, total tannin contents of 0.97 ± 0.004 mg TAE/g CcE, total flavonoid contents of 0.11 ± 0.004 mg QE/g CcE, and total saponin contents of 0.0016 ± 0.0001 mg DE/g CcE.

### 3.2. Quantification of Phenolic Compounds

The identified phenolic and flavonoid compounds in CcE are presented in Table 1, while the respective chromatograms are given in the supporting information (Figure S1).

### 3.3. Phytochemical Analysis by GC-MS

CcE contained 26 different compounds identified through GC-MS, which are presented with retention time, % area, molecular formula, and molecular weight (Table 2). Compounds were identified by using the NIST-11 library, while the GC-MS chromatogram is provided in the supporting information (Figure S2).

### 3.4. C. citratus Inhibits COX and 5-LOX Enzymes

**Figure 1:**
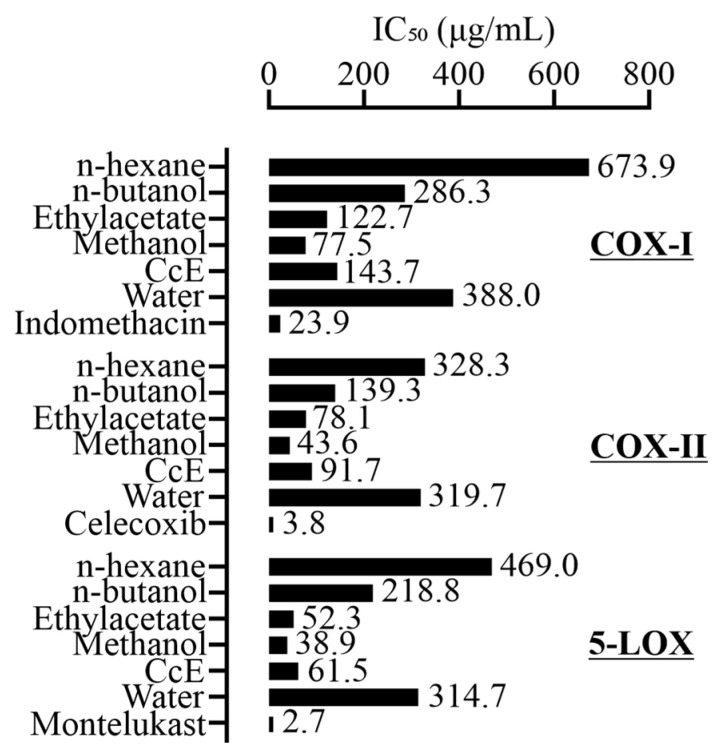
IC_50_ values of *C. citratus* fractions against COX-I, COX-II, and 5-LOX enzymes.

COX-I inhibitory activity was determined for *C. citratus*. fractions (31.25–500 µg/mL). The highest IC_50_ value was observed for n-hexane, followed by water > n-butanol > CcE > ethyl acetate > methanol fraction (Figure 1). Indomethacin was used as a reference for comparison with the IC_50_ of 23.99 µg/mL. The IC_50_ for COX-II was observed in decreasing order, i.e., n-hexane > water > n-butanol > CcE > ethyl acetate > methanol fraction. Celecoxib was used as a reference with an IC_50_ of 3.81 µg/mL. The 5-LOX inhibitory activity was also determined. The IC_50_ for 5-LOX was n-hexane > water > n-butanol > CcE > ethyl acetate > methanol fraction. Montelukast was used as a reference with an IC_50_ of 2.74 µg/mL.

### 3.5. Antioxidant Activity

The total antioxidant capacity of CcE, assessed by the CUPRAC assay, showed EC_50_ values of 0.43 ± 0.04 mg for CcE and 0.24 ± 0.02 mg for ascorbic acid. FRAP assay described the reducing potential of CcE with an EC_50_ value of 4.65 ± 0.07 and ascorbic acid 0.22 ± 0.003 mg. The free radical scavenging activity of CcE was assessed by using DPPH, NO, and H_2_0_2_ scavenging assays. CcE scavenged DPPH with an EC_50_ of 4.56 ± 0.71 mg and ascorbic acid 0.02 ± 0.01mg, respectively. CcE scavenged NO radicals with an EC_50_ of 1.65 ± 0.17 mg and ascorbic acid 0.05 ± 0.004 mg, respectively. In the H_2_0_2_ assay, CcE showed an EC_50_ value of 3.03 ± 0.05 and ascorbic acid 0.41 ± 0.02 mg, respectively.

### 3.6. NOAEL CcE Dose

No signs of toxicity or altered behavior were observed in animals for 14 days; thus, CcE is considered safe for an oral dose of 2 g/kg.

### 3.7. Amelioration of PD Symptoms

**Figure 2:**
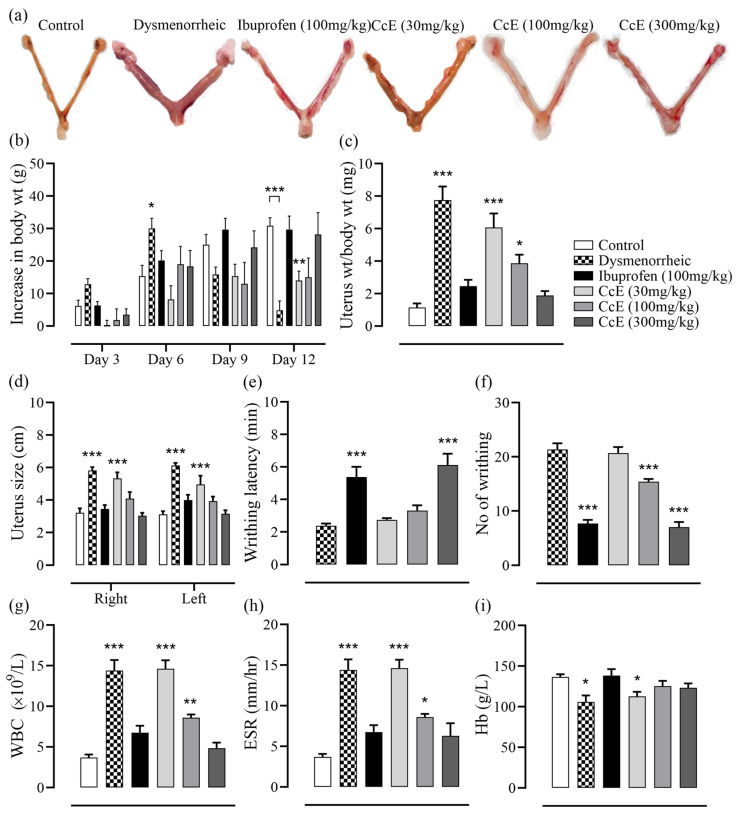
The effect of treatments on different parameters of dysmenorrheic rats: (**a**) representation of the rat uterus; (**b**) changes in body weight; (**c**,**d**) uterus weight and size; (**e**,**f**) writhing latency and total no. of instances of writhing in 30 min; (**g**–**i**) hematological parameters. Treatment groups and dysmenorrheic rats were compared with the normal control group, and significance was determined either by one-way or two-way ANOVA, followed by Dunnett’s multiple comparison test. Data is presented as mean±SEM, where significance is denoted by *** *p* ≤ 0.001, ** *p* ≤ 0.01, * *p* ≤ 0.05 vs. normal control group.

After estrogen treatment, on day 13, animals were injected with oxytocin to induce PD pain. Both the latency time of the first writhing and the total number of writhes were recorded for 30 min. In dysmenorrheic rats, the first writhing appeared at 2.3 ± 0.1 min with a total of 21.3 ± 1.1 instances of writhing. The writhing latency was significantly increased in ibuprofen and CcE 300 mg/kg groups to 5.3 ± 0.6 (*p* < 0.001) and 6.1 ± 0.6 min (*p* < 0.001), respectively. The total number of writhes was also reduced in the ibuprofen, CcE 100, and 300 mg/kg groups (Figure 2e,f). Afterwards, animals were sacrificed for a morphometric analysis of the uterus, which showed increased inflammation and signs of edema in dysmenorrheic, CcE 30, and 100 mg/kg groups. In contrast, animals receiving ibuprofen and CcE 300 mg/kg showed normal uterine tissue morphology (Figure 2a,c,d). Hematological parameters indicated inflammation, with increased WBCs and ESR values in dysmenorrheic, CcE 30, and 100 mg/kg groups. Hemoglobin level was reduced in dysmenorrheic and CcE 30 mg/kg groups (Figure 2g–i). The dysmenorrheic and CcE 30 mg/kg rats were not taking their feed properly, as observed by a minimal increase in body weight on day 12 (Figure 2b).

**Figure 3:**
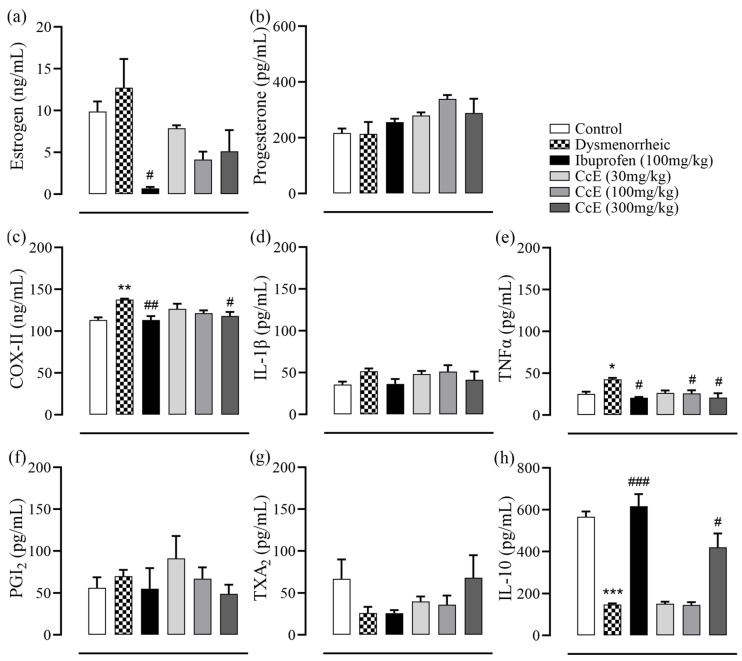
Serum hormone levels: (**a**) estrogen; (**b**) progesterone. Rat uterine tissue inflammatory markers: (**c**) COX-II; (**d**) IL-1β; (**e**) TNFα; (**f**) PGI_2_; (**g**) TXA_2_; (**h**) IL-10. Dysmenorrheic rats were compared with the normal group, while treatment groups were compared with dysmenorrheic rats to assess improvement. Significance was determined by one-way ANOVA followed by Sidak’s multiple comparison test. Data is presented as mean±SEM, where significance is denoted by *** *p* ≤ 0.001, ** *p* ≤ 0.01, and * *p* ≤ 0.05 vs. normal control group; and ^###^ *p* ≤ 0.001, ^##^ *p* ≤ 0.01, and ^#^ *p* ≤ 0.05 vs. dysmenorrheic rat group.

Hormone levels were determined in the rat serum, which indicated that estrogen levels were reduced in the ibuprofen group. Although CcE treatments also showed a decreasing trend compared to dysmenorrheic rats, it was not statistically significant. No significant difference was observed in progesterone levels (Figure 3a,b). Next, we assayed inflammatory mediators from rat uterus tissue homogenate (Figure 3c–h). COX-II levels were significantly increased in dysmenorrheic rats compared to control rats (*p* = 0.01), which were reduced in rats who received ibuprofen and CcE 300 mg/kg treatments. No significant differences were observed in IL-1β, PGI_2_, and TXA_2_ levels. TNFα levels were significantly increased in dysmenorrheic rats (*p* = 0.04), which were reduced in ibuprofen, CcE 100, and 300 mg/kg groups compared to dysmenorrheic rats. The IL-10 levels were reduced in dysmenorrheic rats (*p* < 0.001), whereas they were significantly increased in the ibuprofen and CcE 300 mg/kg groups.

**Figure 4:**
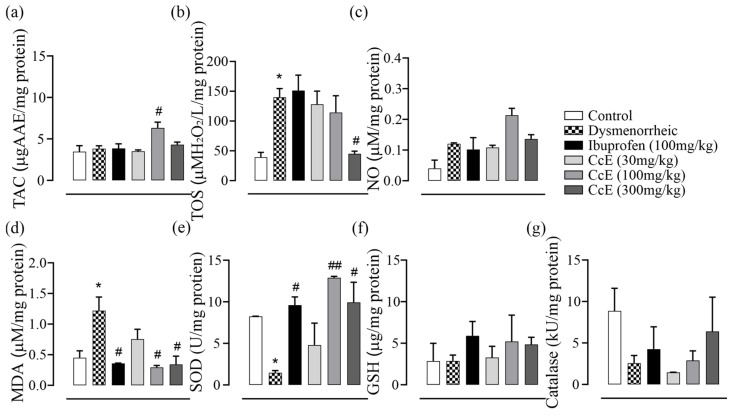
Oxidative stress parameters: (**a**) total antioxidant capacity; (**b**) total oxidative stress; (**c**) NO (**d**) MDA; (**e**) SOD; (**f**) GSH; and (**g**) catalase. Dysmenorrheic rats were compared with the normal group, while treatment groups were compared with dysmenorrheic rats to assess improvement. Significance was determined by one-way ANOVA followed by Sidak’s multiple comparison test. Data is presented as mean±SEM, where significance is denoted by * *p* ≤ 0.05 vs. normal control group and ^##^ *p* ≤ 0.01, ^#^ *p* ≤ 0.05 vs. dysmenorrheic rat group.

Next, we assessed oxidative parameters in rat uterine tissue homogenate (Figure 4a–g). The total antioxidant capacity was increased only in the CcE 100 mg/kg group. Total oxidative stress was increased in dysmenorrheic rats compared to the control (*p* = 0.01), which was reduced in the CcE 300 mg/kg group when compared to dysmenorrheic rats (*p* = 0.02). MDA levels were increased in dysmenorrheic rats (*p* = 0.03), which decreased in ibuprofen, CcE 100, and 300 mg/kg groups compared to dysmenorrheic rats. SOD levels were reduced in dysmenorrheic rats (*p* = 0.03), whereas they were increased in the ibuprofen, CcE 100, and 300 mg/kg groups. No statistically significant difference was observed in NO, GSH, and catalase levels.

**Figure 5:**
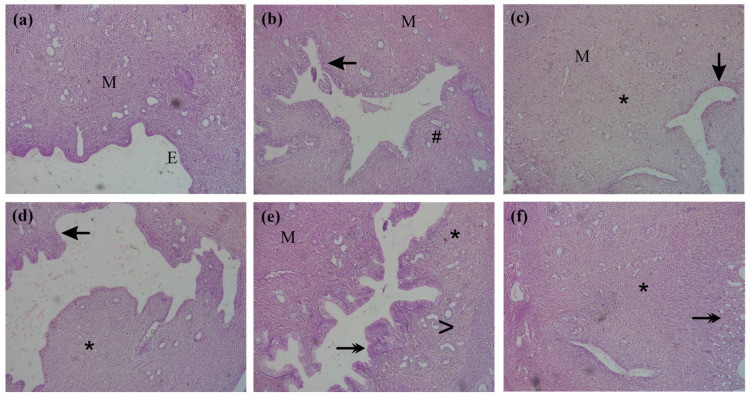
Histomorphology of rat uterine tissue in (**a**) control, (**b**) dysmenorrheic, (**c**) ibuprofen, (**d**) CcE 30, (**e**) CcE 100, and (**f**) CcE 300 mg/kg groups observed at 200× after H&E staining. ‘M’ denotes smooth muscle tissue; ‘E’ denotes endometrium; the arrow sign indicates endometrial hyperplasia and its recovery; a double arrowhead describes endometrial gland atrophy, ‘*’ indicates improved inflammation; ‘#’ indicates inflammatory cell infiltration, and ‘>’ indicates reduction of edema.

The histomorphology (Figure 5a–f) of rat uterine tissue indicated signs of inflammation in dysmenorrheic rats characterized by atypical endometrial and smooth muscle hyperplasia. Endometrial glands also appeared inflamed. Treatment with CcE or ibuprofen improved endometrial hyperplasia and reduced inflammation, accompanied by a decrease in inflammatory cells. Edema was also relieved, and atrophic endometrial glands were observed.

### 3.8. Ex Vivo Uterine Tissue Relaxant Effect

**Figure 6:**
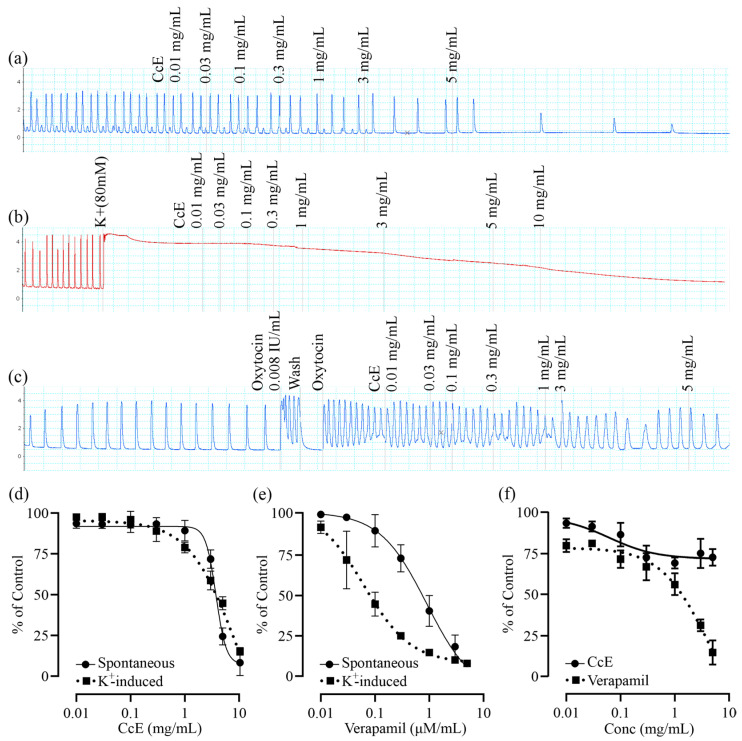
Ex vivo uterine muscle relaxant effect of CcE and verapamil. (**a**–**c**) Representative tracings depicting the uterine tissue relaxant effect of CcE on basal, high K^+^-induced, and oxytocin-induced contractions. (**d**–**f**) EC_50_ values for CcE and verapamil. Each experiment was performed in triplicate, and EC_50_ values were calculated by fitting Hill’s equation.

The effect of CcE was assessed on rat uterine tissue at baseline, high K^+^ (80 mM), and oxytocin-induced contractions. CcE (0.01–10 mg/mL) relaxed basal spontaneous contractions with EC_50_ of 3.76 mg/mL (95% CI: 3.36–4.24 mg/mL). CcE also relaxed high K^+^-induced sustained contractions with EC_50_ of 8.74 mg/mL (95% CI: 5.16–16.71 mg/mL). To assess CcE efficacy in dysmenorrhea, contractions were induced in the uterus tissue of female rats with a synchronized estrous cycle. CcE moderately relaxed the contracted tissue at 5 mg/mL, which was not sufficient to calculate EC_50_. Verapamil was used as a standard drug for comparison (Figure 6).

### 3.9. Analgesic Effects of CcE

**Figure 7:**
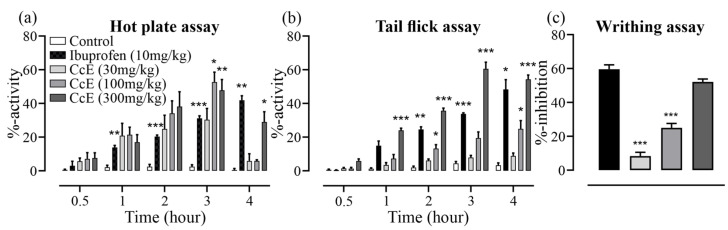
Analgesic effect of CcE and ibuprofen: (**a**) hot-plate assay; (**b**) tail-flick assay; (**c**) acetic acid-induced writhing assay. Significance was determined by two-way ANOVA followed by Tukey’s multiple comparison test. Data is presented as mean±SEM, where significance is denoted by *** *p* ≤ 0.001, ** *p* ≤ 0.01, * *p* ≤ 0.05 vs. normal control group.

In the hot-plate assay, CcE 100 and 300 mg/kg reduced pain sensation at the 3rd hour (*p* = 0.03 & 0.009, respectively) while the analgesic effect of CcE 300 mg/kg persisted until 4 h (*p* = 0.04) compared to control (Figure 7). In the tail-flick assay, CcE 300 mg/kg showed a significant analgesic effect compared to control starting from the 1st hour (*p* < 0.001), which persisted until 4 h (*p* < 0.001). The analgesic effect of CcE 100 mg/kg was observed starting at the 2nd hour (*p* = 0.02), and persisted until 4 h (*p* = 0.04), while CcE 30 mg/kg did not exhibit any analgesic effect. In the acetic acid-induced writhing assay, CcE 300 mg/kg inhibited 52.0 ± 1.7% instances of writhing and was equivalent to ibuprofen 10 mg/kg, which inhibited 59.5 ± 2.6% instances of writhing. CcE 100 and 30 mg/kg inhibited 25.0 ± 2.5 and 8.3 ± 2.2% instances of writhing, respectively (Figure 7c).

## 4. Discussion

Dysmenorrhea is a significant health concern in adolescent medicine, affecting the quality of life in young adults. The first-line treatment of PD is NSAIDs, relieving symptoms by inhibiting PGs synthesis, thus reducing the cramps and associated pain. Other pharmacologic options include hormonal contraceptives, nitric oxide, magnesium, and calcium channel blockers. The non-pharmacologic measures include lifestyle modifications, acupuncture, and the use of herbs, for instance peppermint, lemon balm, cinnamon, fennel, and ginger [25].

*Cymbopogon citratus* (DC.) Stapf is used in folkloric medicine for its analgesic, anti-inflammatory, anti-spasmodic, and antioxidant properties, targeting menstrual disorders [10]. Despite these contributory activities, the plant has not been evaluated for its efficacy in managing PD-associated discomfort. We investigated its effectiveness in the PD rat model, and explained its mode of action through antispasmodic effects on rat uterine tissue, anti-inflammatory activity due to the inhibition of cyclooxygenase and lipoxygenase enzymes, and analgesic effects.

The aerial parts of *C. citratus* were extracted with solvents in increasing polarity. The prepared fractions were first assessed for their COX-I, COX-II, and 5-LOX inhibitory potential (Figure 1). The ethyl acetate, methanol, and 70% methanol fractions exhibited potent inhibition of all three enzymes compared to the other fractions. For further experiments, we selected a 70% methanol fraction designated as CcE for two reasons. First, it inhibited the COX-I enzyme less potently compared to ethyl acetate and methanol fractions. Secondly, it demonstrated the highest yield compared to the other two fractions. Next, we performed a phytochemical quantification of CcE, which indicated good phenolic (1.75 ± 0.03 mg GAE/g CcE) and flavonoid (0.11 ± 0.004 mg QE/g CcE) contents. To assess non-polar phyto-constituents, the CcE was subjected to GC-MS analysis, which identified 29 compounds from different chemical classes (Table 1). Among them, several compounds are known for their anti-inflammatory effects. For instance, n-hexadecanoic acid competitively inhibits phospholipase A_2_, thus subsequently suppressing the inflammatory cascade [26]. Linoleic acid is anti-inflammatory [27] and known to relax PGF2α pre-contracted smooth muscles of coronary arteries by acting on Na^+^/K^+^ ATPase [28]. Oleic acid is anti-inflammatory and antioxidant [29]. Erucic acid is used as a dietary supplement for cognitive improvement, as well as its antioxidant and anti-inflammatory effects [30].

The presence of anti-inflammatory, anti-spasmodic, analgesic, and antioxidant compounds complement each other, thus providing beneficial ameliorating effects in PD-associated discomfort, as observed in animal experiments. The administration of oxytocin after 12 days of estrogen treatment induced PD pain in animals, which was observed in the form of abdominal contortions. Both writhing latency and total number of writhes were reduced in the groups treated with CcE 100 and 300 mg/kg compared to dysmenorrheic rats with increased latency (Figure 2e,f). This symptomatic response was associated with improvements in uterine tissue morphometric features and histology (Figure 2a,c,d and Figure 5), which showed resolved edema and reduced inflammation. COX-II, TNFα, IL-10 levels, and oxidative stress parameters were also improved (Figure 3c–h and Figure 4). The rat serum hormones showed a reduction of estrogen levels with ibuprofen treatment, but no significant difference was observed in the CcE treatment groups for estrogen and progesterone levels.

The mechanism of pain alleviation was further assessed by ex vivo uterine tissue experiments (Figure 6), where CcE relaxed basal and high K^+^-induced contractions in a dose-dependent manner. However, it modestly relaxed oxytocin-induced contraction, suggesting that anti-spasmodic action is not the sole mechanism of pain alleviation. So, we assessed the analgesic effect of CcE in mice using tail-flick, hot-plate, and acetic acid-induced writhing experiments (Figure 7). CcE 300 mg/kg significantly reduced pain sensation in all three assays [31]. We used the same dose range of CcE in mice (30, 100, and 300 mg/kg) as was applied to rats for the management of dysmenorrhea. We did not apply dose conversion for mice, which would have been (15, 50, and 150 mg/kg). So, this may be considered a limitation of the study.

CcE contained several phenolic and flavonoid compounds (Table 1), and it showed good antioxidant activity. CcE is also nutritionally rich and contains proteins, carbohydrates, fibers, vitamins, and minerals [32]. Several volatile oils present in lemongrass make it possess a pleasant flavor; hence, it is used in cuisine and herbal tea. The presence of analgesic, anti-inflammatory, and spasmolytic activities relieves dysmenorrhea-associated symptoms and makes it a good candidate for consumption in herbal tea. CcE 300 mg/kg was effective in reducing the dysmenorrhea symptoms in rats, which is equivalent to 1.7 g of dried *C. citratus* leaves. The human (60 kg) equivalent dose is 1.4 g of dried *C. citratus* leaves, which may be consumed in the form of an herbal tea.

## 5. Conclusions

*C. citratus* is a popular herbal tea consumed by several communities. It inhibits COX-I and COX-II enzymes and relieves primary dysmenorrhea-associated symptoms through its analgesic, anti-inflammatory, and antispasmodic activities, which complement each other to relieve pain.

## Figures and Tables

**Table 1:** Phenolic, flavonoid, and other compounds identified in CcE by reversed-phase HPLC analysis.

Compound	Retention Time (min)	Area (%)	Quantification (ppm)
Quercetin	2.64	0.5	1.41
Gallic acid	4.46	19.1	36.82
Caffeic acid	12.43	2.2	5.69
Vanillic acid	13.36	2.0	6.58
Benzoic acid	14.70	2.3	13.12
Syringic acid	16.86	1.9	2.52
p-Coumaric acid	17.92	2.0	1.39
m-Coumaric acid	19.78	2.4	1.55
Ferulic acid	22.43	1.0	3.79
Cinnamic acid	25.34	1.1	2.15
Sinapic acid	26.20	0.7	0.45
Kaempferol	9.1	69.9	16.5

**Table 2:** Compounds identified in the GC-MS analysis of CcE.

Peak No.	RT (min)	Area (%)	Compound	M.F.	M.W. g/mol	Qual
1	17.2	1.3	n-Hexadecanoic acid	C_16_H_32_O_2_	256.4	99
3	19.1	22.8	10E, 12Z-Octadecadienoic acid	C_18_H_32_O_2_	280.4	97
4	19.2	11.3	Oleic acid	C_18_H_34_O_2_	282.5	99
5	19.3	2.0	Octadecanoic acid	C_18_H_36_O_2_	284.5	99
6	19.4	1.3	linoleic acid	C_18_H_32_O_2_	280.4	99
7	19.7	0.6	Isolinoleic acid	C_18_H_32_O_2_	280.4	99
8	20.2	0.4	2-Octylcyclopropaneoctanal	C_19_H_36_O	280.5	93
9	20.8	9.8	cis-11-Eicosenoic acid	C_20_H_38_O_2_	310.5	99
10	21.0	0.9	Eicosanoic acid	C_20_H_40_O_2_	312.5	95
11	21.2	0.1	9, 17-Octadecadienal, (Z)-	C_18_H_32_O	264.4	96
12	21.3	0.2	1-cis-Vaccenoylglycerol	C_21_H_40_O_4_	356.0	99
13	21.6	1.6	Linoelaidic acid	C_18_H_32_O_2_	280.4	92
14	21.7	3.6	Glycidyl oleate	C_21_H_38_O_3_	338.5	99
15	21.9	0.9	Gadoleic acid	C_20_H_38_O_2_	310.5	58
16	22.0	0.3	2-Hydroxycyclopentadecanone	C_15_H_28_O_2_	240.38	96
17	22.5	20.4	Erucic acid	C_22_H_42_O_2_	338.6	99
18	22.6	1.2	18-Nonadecenoic acid	C_19_H_36_O_2_	296.5	97
19	22.9	0.2	Z, E-2,13-Octadecadien-1-ol	C_18_H_34_O	266.5	93
20	23.1	0.3	Elaidic acid	C_18_H_34_O_2_	282.5	93
21	23.3	2.6	Glyceryl monolinoleate	C_21_H_38_O_4_	354.5	96
22	23.3	5.3	Glyceryl monooleate	C_21_H_40_O_4_	356.5	95
23	23.5	0.2	Glyceryl monostearate	C_21_H_42_O_4_	358.6	99
24	23.9	0.2	bis(2-ethylhexyl) benzene-1, 4-dicarboxylate	C_24_H_38_O_4_	390.6	87
25	24.6	0.4	Erucoyl chloride	C_22_H_41_ClO	357	55
26	25.2	2.5	11-Eicosenoic acid, methyl ester	C_21_H_40_O_2_	324.5	46
29	28.2	5.8	Monoerucin	C_25_H_48_O_4_	412.6	64

## Data Availability

Data will be available on reasonable request.

## Supplementary Materials

The following supporting information can be downloaded at: https://www.mdpi.com/article/10.3390/antiox14070838/s1, Figure S1: HPLC chromatogram of CcE; Figure S2: GC-MS chromatogram of CcE.
