# Supplementary material for: Lemongrass Alleviates Primary Dysmenorrhea Symptoms by Reducing Oxidative Stress and Inflammation and Relaxing the Uterine Muscles
Source: Antioxidants (Basel). 2025 Jul 8;14(7):838. doi: 10.3390/antiox14070838 (PMC12291959; doi:10.3390/antiox14070838)
Supplement: Supplementary file 1 [file antioxidants-14-00838-s001.zip › antioxidants-3637314-supplementary.pdf]

Lemon Grass Alleviates Primary Dysmenorrhea Symptoms by Reducing Oxidative Stress and Inflammation and Relaxing the Uterine Muscles

Sheikh Safeena Sidiq [1], Qaiser Jabeen [1], QurratUlAin Jamil [2], Muhammad Saeed Jan [3], Iram Iqbal [4], Fatima Saqib [4], Mohammed Aufy [5,*] and Shahid Muhammad Iqbal [1,6,*]

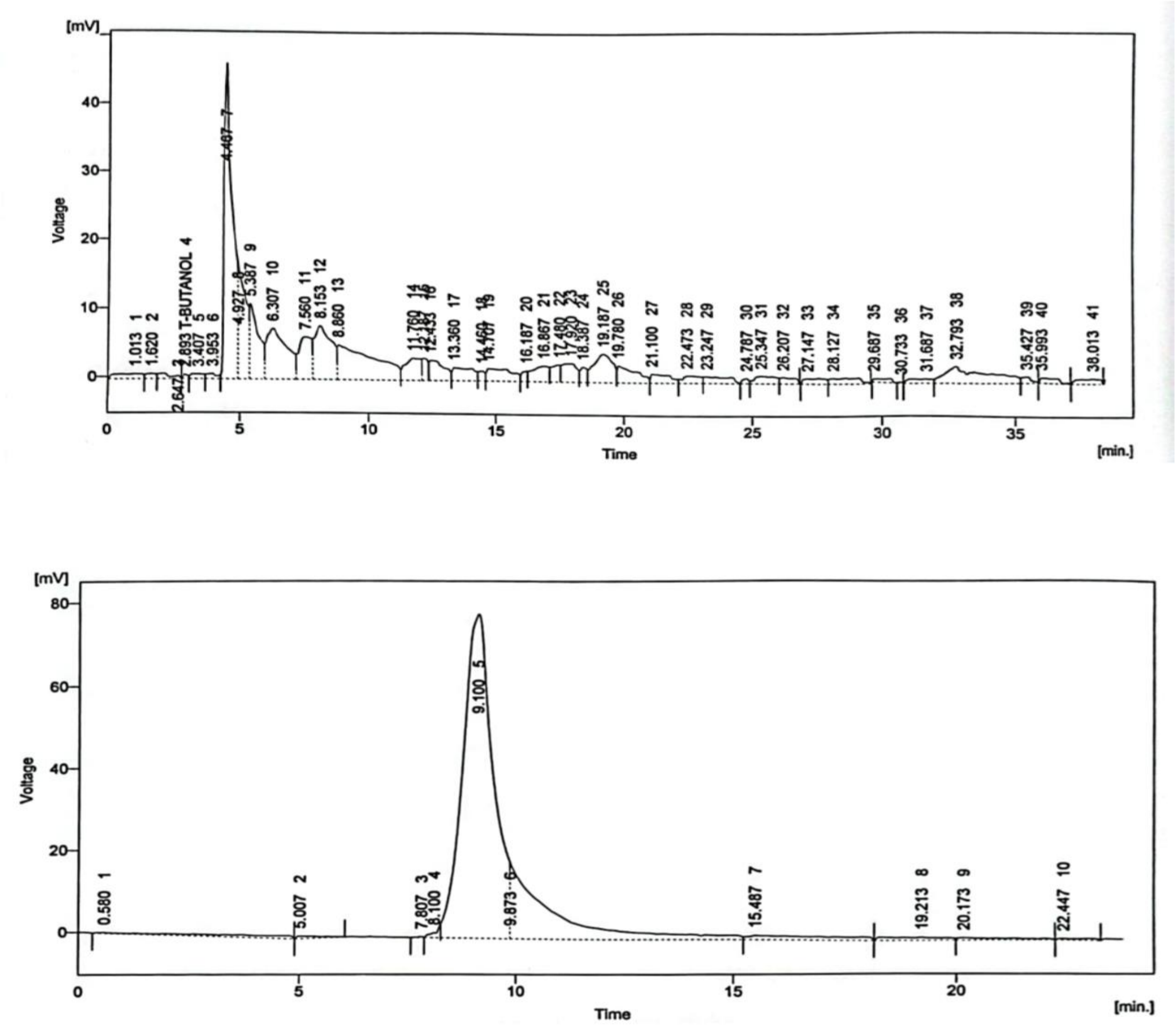

FIGURE S1. HPLC chromatogram of CcE showing peaks and retention time of identified phenolic and flavonoid compounds.

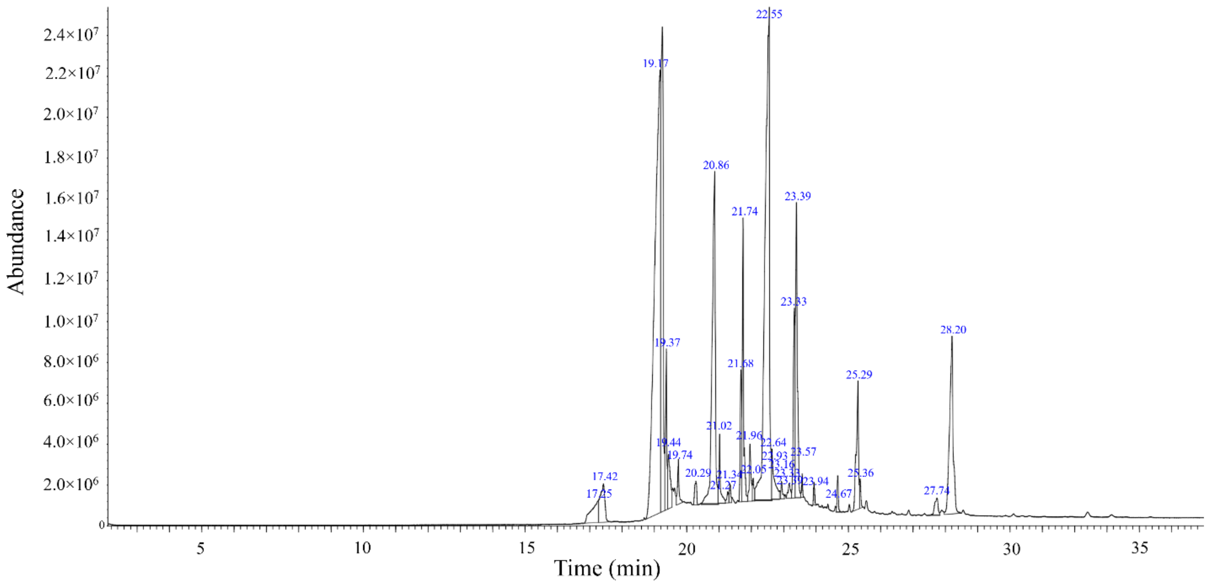

Figure S2. GC-MS chromatogram of CcE showing peaks of various identified compounds.
